# Commentary on “The role of domain-general cognitive control in language comprehension” by Fedorenko

**DOI:** 10.3389/fpsyg.2014.00629

**Published:** 2014-07-02

**Authors:** David Caplan

**Affiliations:** Massachusetts General HospitalBoston, MA, USA

**Keywords:** executive function, language localization, modularity, language localizer, functional architecture

Fedorenko (2014) examines “the relationship between high-level language processing and domain-general cognitive control, with a focus on the *brain systems* that support these cognitive capacities (ital hers).” She addresses two questions –“(i) when (i.e., under what circumstances) the cognitive control mechanisms get engaged during language understanding; and (ii) whether this engagement is necessary for comprehension.” The first part of Fedorenko's paper presents data that she says shows that (1) a neural “language system” is stable within and across individuals, time, modality of presentation, and language, and that (2) a “multiple-demand (MD) system” that is activated by tasks that contrast conditions that vary in difficulty (an executive control system) is anatomically separate from the language system. This forms the backdrop to a far-ranging discussion of the two questions in the second part of the paper. As Fedorenko's emphasis is on the contribution of neural data to answering these questions, my commentary will focus on the relevance of the BOLD signal data reported in Part 1 to them. I note at this point that there are issues about the results Fedorenko presents in Part 1 of her paper. For instance, the within-subject stability of the activations produced by Federonko's language localizer across time is documented in only four individuals—higher than the industry standard of zero for the number of participants tested twice for a BOLD signal effect, but not a large number. However, my comments will accept Fedorenko's results and ask what they show.

There are two functional neuroanatomical findings reported in Section 1 of Fedorenko's paper that could bear on her questions. The first is the finding that tasks that activate the MD/executive system do not activate the language system. I do not think this result is relevant to the questions posed. The crucial question is whether the executive control system needs to activate the comprehension functions localized by Fedorenko's language localizer when it performs executive functions in the tasks that Fedorenko used to localize it. As far as I can see, it does not. Fedorenko localized the high level language comprehension system by subtracting BOLD signal associated with item recognition in lists of non-words from that associated with recognizing words in sentences (and, separately, by contrasting passive listening to these stimuli), and localized the MD system by varying complexity of arithmetic processing and working memory and with the Stroop task. The executive system does not regulate operations that perform high level language comprehension when it is involved in arithmetic processing and Stroop (whether it does so when it performs WM tasks is a matter of debate; Caplan and Waters, 1999, 2013). In Stroop, for example, the executive functions needed in the interference condition and not in the baseline conditions involve lowering activation of the word derived from print relative to that of the name of the color in order to select a word for production. The aspect of language processing that is regulated is the word production system (Roelofs, 2008), not high level comprehension. The fact that Stroop does not activate areas identified by Federorenko's language localizer therefore does not imply that executive functions do not control language operations in language comprehension tasks.

The second finding that might inform the questions Fedorenko poses is the reverse aspect of the non-overlap—that Fedorenko's language localizer does not activate the MD system. However, this direction of inference also fails. Power aside, the absence of a difference in BOLD signal in executive areas in the contrast of an experimental and a baseline task does not show that executive functions are not needed in the experimental task; it only shows that they are not used to a greater extent in the experimental than in the baseline task (Caplan, 2009). The absence of an effect of the [sentence-nonword] condition on MD areas is entirely consistent with the MD/executive system performing operations critical to high level comprehension but performing the same operations, or ones that require the same degree of executive control, in the baseline task.

Turning to more general issues, there is an important gap in Fedorenko's discussion—she does not present a framework for understanding the relation between executive control operations and the operations performed by domain-specific processors in the performance of a task. Further consideration of the effects of the language localizer on BOLD signal in MD areas shows why such a model is needed. Fedorenko found that the [nonword-sentence] contrast activated MD areas. We need a model of how executive controls operates in these two tasks to understand why this happened. I will outline a very simple model, and suggest how it may account for this finding.

Work by many researchers (e.g., Meyer and Kieras, 1997; Engle, 2002) has led to the view that the executive system sets and maintains task goals, activates the components of the functional architecture needed to perform a task, directs attention to the stimuli that those components operate on, and monitors response selection for consistency with task goals. With respect to these functions in the conditions in Fedorenko's localizer, setting and maintaining task goals (to recognize probes in the memory version of the localizer; unspecified goal in passive listening), directing attention to the stimuli, and monitoring response-goal compatibility are required, and arguably the same, in both conditions. The difference in executive functioning in the two conditions lies in the functional architecture that the executive control system activates. In the sentence condition, it presumably activates operations that recognize words; in the non-word list condition, it activates operations that create representations of non-words. It also activates mechanisms that retain these percepts in STM. While it is clear that sentence comprehension mechanisms operate in the sentence condition, it is not clear that they are activated by the executive control system; they may be automatically activated by attending to the input in order to recognize words for later recall. However, it is also possible that the executive control system activates both sentence comprehension operations and those that recover sentence form from meaning (Lombardi and Potter, 1992), because this may be the easiest way to meet task goals. Regardless, activating any of these functional architectures in the sentence condition is arguably easier than activating the operations that identify and retain non-words in the list condition, due to the immense practice comprehenders have with the first set and their almost complete unfamiliarity with the second. This would explain the finding of activation of MD areas by the non-word list compared to the sentence conditions.

My point so far is that BOLD signal is no different than behavioral variables in one important respect—we need models of the role of executive operations as well as domain-specific operations in performing all conditions of the tasks used in an experiment to interpret BOLD signal in MD and language areas in a contrast of two conditions. Non-overlap or reversed effects of conditions may be expected given certain models of the role of executive control in regulating domain-specific operations in the tasks used in some studies. A second point, with which I will conclude, pertains to the well-recognized and oft cited temporal (in)sensitivity of BOLD signal.

BOLD signal has a resolution of several seconds. Some executive control operations that differ between conditions in fMRI studies likely take place for a small fraction of the time over which neural activity affects BOLD signal in a condition. For example, task-switching studies suggest that, if conditions are blocked, activating the functional architecture needed in a condition (the executive function that I suggested differed in the two conditions in Fedorenko's language localizer) might be performed once at the outset of a block of trials in that condition (Monsell, 2003), leading to a very small contribution of activity of the executive control system on BOLD signal in a condition. Non-activation of a brain area in a contrast of two conditions may thus result from the insensitivity of BOLD signal to transient cognitive operations. To capture the neural correlates of these short-lived operations requires measuring neural activity on the time scale at which they apply, in the areas where they apply. This requires measures such as MR-constrained MEG/EEG signal. When we have used this measure, we have consistently found activity in MD areas of the brain that interacts with activity in language areas of the brain during on-line sentence comprehension (unpublished data). The interpretive challenge in our studies has been the extent and the extensive pattern of interactions of EEG/MEG activity seen during on-line comprehension.

To conclude, Fedorenko has taken on the great challenge of using neural data to resolve, or at least influence the answer to, one of the most debated questions in cognitive science and psycholinguistics: how control operations interact with domain-specific operations in higher level language comprehension. I suggest that this effort requires consideration of cognitive models at a least a moderate grain size. It may also require use of measures of neural activity that have significantly better temporal resolution than BOLD signal.

## Conflict of interest statement

The author declares that the research was conducted in the absence of any commercial or financial relationships that could be construed as a potential conflict of interest.
